# Considerations for Monitoring School Health and Nutrition Programs

**DOI:** 10.3389/fpubh.2021.645711

**Published:** 2021-07-16

**Authors:** Linda Schultz, Julie Ruel-Bergeron

**Affiliations:** Global Financing Facility, Health, Nutrition and Population Global Practice, World Bank, Washington, DC, United States

**Keywords:** monitoring, adolescent health, school feeding, school closure, multisector investments, human capital, school health and nutrition, adaptive programming

## Abstract

School health and nutrition (SHN) interventions are among the most ubiquitous public health investments and comprise a key mechanism for reaching populations that are otherwise difficult to reach through the health system. Despite the critical role of monitoring these multisectoral programs to enable data-informed adaptive programming, information to guide program implementers is scant. This manuscript provides an overview of how monitoring indicators can be selected across a SHN program's logical framework, with specific examples across five SHN implementation models. Adaptation of SHN programs in times of school closures, such as those currently being experienced globally due to the emergence of COVID-19, is also addressed. Key aspects of SHN program monitoring are explored, including: (1) why monitor; (2) what to measure; (3) how to measure; and (4) who measures. In situations of school closures, strategies to shift both program activities and corresponding monitoring mechanisms are critical to understanding the rapidly evolving situation and subsequently guiding policy actions to protect vulnerable populations.

## Introduction

School health and nutrition (SHN) interventions are among the most ubiquitous public health investments worldwide, with more than 100 countries offering school-based or school-linked routine health and nutrition services (1). SHN has become a common intervention due to the broad recognition that healthy students learn better. In addition, school-based service delivery is compelling to both the health and education sectors: this approach reaches populations that traditionally have little contact with health facilities and targeted health interventions create a more level playing field for vulnerable students to benefit from the existing investments in education (2). Although SHN services have historically targeted primary schools, variations in age at school entry and late enrollment enable this platform to reach at a minimum, young adolescents as well (3). In addition, rising rates of secondary school enrollment globally (4) make the school platform yet again an attractive mechanism for reaching adolescents with health and nutrition services as they enter critical periods of physical and socio-emotional development, and sexual debut.

Well-designed SHN interventions align with critical periods of development and address social determinants of health for school-age children and adolescents (5). SHN interventions can include routine health service delivery and should be complemented with improved water, sanitation, and hygiene (WASH) infrastructure, and related messaging within existing curricula. The benefits and breadth of SHN interventions to improve human capital formation have been well-summarized elsewhere (1, 2, 6). Delivering school health services alone, however, is not sufficient to improve health or learning outcomes; rather, the quality, consistency, and relevance of services and delivery modalities is critical to achieve their intended outcomes. Furthermore, the COVID-19 pandemic has revealed the importance of having “back-up mechanisms” for the delivery of school health and nutrition services that can quickly pivot to meet the needs of school-going children and adolescents in periods of prolonged school closures.

As with any development program, it is important to monitor the implementation of SHN services, particularly as these represent significant investments. As an example, an estimated USD 41–43 billion is spent annually on school feeding programs alone (7). Program monitoring is deeply engrained in programmatic preparation and implementation, serving as a tool to track implementation progress against its goals through frequent or routine collection of process and output-based indicators throughout the life of the project's implementation. To set the stage for program monitoring, implementers should agree on: (1) the problem the project is trying to solve; (2) how the project inputs will lead to desired outcomes; (3) the type of evidence needed to assess progress toward program results; and (4) the existing data sources and instruments available in the country.

There are varying approaches to deliver and monitor school health and nutrition services, and the range of relevant interventions to deliver can vary widely across contexts. As a result, there is no blueprint for delivering and monitoring high quality and equitable SHN programming and each monitoring system will be unique to the programs, interventions, and context. Carefully selected and measurable indicators help project planners and implementers assess intervention quality, inform mid-course revisions where needed, and ensure resources are being used effectively.

The multisectoral nature of SHN interventions requires further careful planning and coordination to meaningfully capture monitoring data, as there are two or more, as is often the case for school feeding, sectors and stakeholders (such as parents and pupils themselves) to engage. This principle remains true even for monitoring interventions that have long engaged multiple sectors for school-based delivery, such as deworming, as there may be limited coordination between information systems to facilitate the transfer of information from one to the other. These governance and accountability challenges mean that countries may have limited ability to monitor implementation progress to inform future service delivery (8).

This manuscript presents key principles for SHN program monitoring and provides broad guidance aimed at supporting the development and implementation of monitoring mechanisms within SHN programs. The discussion includes specific examples of indicators used to monitor the delivery of SHN interventions and considers monitoring responsibility and information flows between education and health sectors at the national, subnational, and individual level. Emphasis is also placed on governance and accountability of monitoring processes, such as responsibility of collecting data, and information flows between the health and education sectors to maximize data-informed decision-making. Although program monitoring is often complemented by program evaluation, this manuscript focuses on monitoring, given the paucity of information on the topic.

## Monitoring SHN in Countries and Innovation During and Following Prolonged School Closures

### Monitoring Responsibility

**Figure 1:**
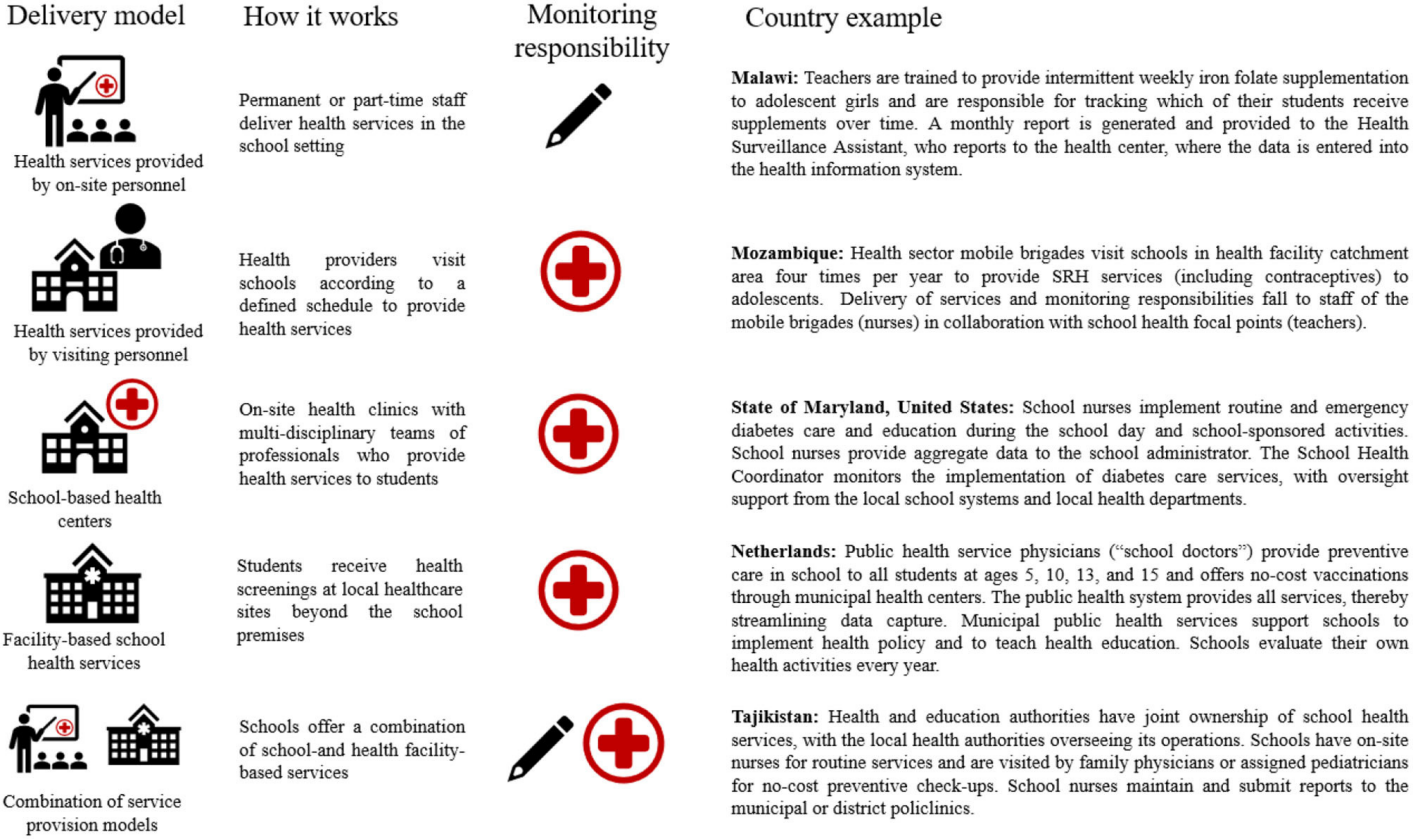
Information flow between schools and ministries by school health and nutrition delivery modality. The encircled cross icon represents the health sector and the pencil icon represents the education sector. Information for Malawi and Mozambique were contributed based on personal communication with World Bank staff. Sources for the summary on Maryland, United States: (9); Sources for the Netherlands: (3, 10–13); Sources for Tajikistan: (10, 13–15).

Data collection for SHN services are often led by either the education or health sector. Baltag and colleagues categorized five delivery modalities for SHN services (1), with monitoring responsibilities typically aligning with the cadre who are providing the intervention as presented in Figure 1. For example, in a delivery model where health services are provided by on-site personnel, the monitoring responsibility falls to those cadres (educators/teachers), who then must produce monthly reports of service delivery to front line health workers who push the information up the health management information system (HMIS). In delivery models that involve health service delivery by visiting or permanent health staff, the monitoring and reporting responsibility is taken on fully by the health sector, with a communication channel extended to the education sector. Alternatively, collection methods may reflect the sector-specific technical considerations for delivery of standalone interventions, for example in the case of HPV vaccine delivery, which is almost exclusively delivered and monitored by the health sector.

### The Role of Monitoring in Program Adaption

Program monitoring is just as important for documenting the reach of an intervention as it is for identifying when adjustments to the service delivery design are necessary to maximize their intended impact. For this reason, program monitoring should include a feedback loop that facilitates the flow of information from the subnational to the national level and vice versa, to guide policies and enable adaptive programming that promotes course correction. Table 1 provides a granular summary on the process select countries take when collecting and reporting school health and nutrition data, and includes information when data is moving from schools to the lead ministry. The summaries captured within both Figure 1 and Table 1 are specific to the data collection and reporting process for these programs, and it is important to note that the roles for delivering and collecting data can vary within and across countries depending on the program design.

**Table 1:** Monitoring considerations and methods for selecting ASHN interventions.

**Country**	**Data collection and reporting mechanisms**
**Deworming**	
Kenya	Following Deworming Days, schools send completed monitoring forms to their division/ward-level Area Education Officer, who then compiles and shares collated data with the Sub-County Directors of Education, who in turn, submits sub-county-level summaries to the Sub-County Medical Officers of Health and to the National Office for Data Analysis and Financial Management. Finally, school-based deworming data is captured within the HMIS (16).
**IFA supplementation**	
Bhutan	The Ministry of Education requests that schools submit regular reports on the delivery of IFA supplementation to adolescent girls and boys. Teachers provide a report to the principal, who in turn collates the school's data and shares it with the District Health Officer, the District Education Officer, the School Health Program at the MOH, the Food and Nutrition Program at the MOH, as well as with the Comprehensive School Health Division at the MOE (17).
**School feeding**	
Ecuador	Ecuador has an information management system that provides real-time information to the national program on the number of breakfasts and snacks delivered and school children reached. School Food Program officials use this system to monitor program implementation and to intervene when programs are underperforming. In addition, the national budget includes a line item specifically for monitoring school feeding implementation (18).
Tanzania	The Tanzania Education Management Information System (EMIS) includes metrics related to school feeding (19), such as the source and amount of financial contribution (central government, council level, contribution by community, etc.) for school feeding. Data for the EMIS is collected through the Annual School Census every year and is digitized and stored at the national level.
**School health policy**	
Lao PDR	The National School Health Policy (NSHP) spans five components: personal health and life skills, healthy school environment, health and nutrition services, control and prevention of common diseases, and school and community partnership. Province and district educational offices monitor and report on the implementation of the NSHP to the Ministry of Education and Sports (20).
**Vision screening**	
Pakistan	Pakistan conducts school-based vision screening with support from Sightsavers International. Trained teachers in participating schools conduct vision screening of all school-attending children and other teachers, document screening results in a standardized form, and submit forms to the school principal. Principals collate and submit school-wide forms to the Designated Education Officer at the Local Education Department, who in turn, submits district-level data to the Health Department indicating where follow-up is needed. In parallel, participating hospitals accept referrals from the school vision screening program and submit monthly status reports to the National Program for Prevention and Control of Blindness as well as with the Sightsavers Pakistan Country Office. Sightsavers consolidates all programmatic data and submits quarterly reports to the Federal Directorate of Education at the Ministry of Education.

*Information for vision screening was collected through personal communication with Sightsavers International*.

Though not specific to SHN programming, a successful example of adaptive programming based on a well-functioning feedback loop was seen in Malawi, when a community-based nutrition program successfully adapted its programming by using real time monitoring data, adding service delivery sites in areas with low attendance, following-up with participants who had repeated absences, and improving targeting of services to improve procurement efficiencies and achieve cost savings (21). Monitoring of school-based programming can similarly use real-time data to identify whether there are trends among students who have routinely missed the delivery of interventions and serve as the starting point for reconsidering how best to reach those individuals going forward.

Pandemics, such as the one facing the world in 2020, and other types of socio-political or environmental crises that cause school closures, have further demonstrated the utility in adapting both the service delivery mechanism and the modality for collecting monitoring data in extenuating circumstances. Emerging evidence from the COVID-19 pandemic suggest that public health emergencies make accessing social services more difficult, and this is particularly true for those who depend on the education system as their delivery mechanism. An unprecedented number of countries worldwide closed schools to slow the transmission of COVID-19, with more than 192 countries mandating some form of school closures, impacting at least 1.6 billion children and youth and an estimated 63 million teachers (22, 23). In response, program implementers have pivoted service delivery to continue the provision of health services that were previously school-based to reach vulnerable populations.

School meals and cash transfers constitute one example: recognizing that school meals provide immediate benefit for undernourished students, Thailand began to distribute shelf-stable milk to the homes of students who would otherwise receive meals in schools (24); Guatemala engaged Parent-Teacher Associations to deliver 2-week lunch rations (25); and the World Food Programme piloted digital food vouchers, contactless cash transfers, and delivery of take-home rations to families, among other approaches (26). India, which provides the largest school feeding program worldwide, similarly adapted its delivery and monitoring mechanisms. In Uttar Pradesh State, schools provided grains for over 75 days, directly transferred a stipend for cooking costs to beneficiary bank accounts, and used an interactive voice response system to confirm that both the funds and foodstuffs were received. Meghalaya State, on the other hand, engaged District School Education Officers to confirm that parents had received their entitlements for the duration of school closures. Schools in Meghalaya State retained the data and submit reports to their respective District School Education Officers, Sub-Divisional School Education Officers, and the Directorate of School Education and Literacy. Data collected through these various monitoring mechanisms suggest that despite each states' best efforts, the alternative delivery platforms reach only half of its intended beneficiaries (27). This data is valuable in that it guides program implementers in each state on which populations need additional and varied forms of targeted outreach to ultimately benefit from the provisions provided by their respective states.

## Key Program Elements: Indicators, Reporting, and Monitoring Mechanisms

As discussed, given variations in program design, implementation, and monitoring, this manuscript emphasizes key principles to designing robust and effective monitoring systems, as opposed to prescriptive guidance on specific indicators or monitoring mechanisms. These principles include: (1) Selection of indicators to adequately measure the intended final impact and identification of appropriate data sources; (2) simplicity and feasibility for data collectors; (3) integration of monitoring data into other sources of information; and (4) clarity in all stages of the data collection, reporting, analysis, referrals and feedback processes. This section provides an overview of how to apply these principles to the specific considerations that program implementers must weigh when monitoring a multi-sector effort, such as a SHN program.

### Indicators to Monitor School Health and Nutrition Programming

**Figure 2:**
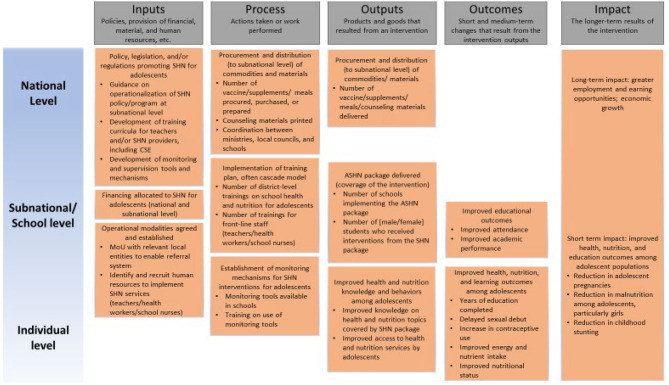
Illustrative logical framework for adolescent school health and nutrition interventions.

Indicators drive resource allocation as well as all subsequent data collection, analysis, and reporting. As with the design and implementation of any program or project, the selection, monitoring, and validation of appropriate indicators specified within the results framework is critical to incentivizing not only the inclusion, but also the implementation of SHN services. The selection of specific indicators that make up the results framework are largely dependent on and closely aligned with the program's logical framework. Figure 2 provides an illustrative example of a program logical framework and a broad set of actions that could be considered in SHN programming.

In a country programmatic scenario, this logical model would require adjustments based on the specificity of interventions included in the basic package, activities needed to deliver them, and intended results of those. Indicators can then be categorized according to which programmatic process they measure along the program's logical framework, such as inputs, processes, outputs, outcomes, and impacts (refer to Figure 2). The selected indicators will subsequently serve as the basis for the programmatic monitoring plan.

The wide variation in SHN programs and delivery models render it difficult to be prescriptive about specific indicators to use. Table 2 provides an illustrative example of a limited number of proposed indicators under the categories of processes traditionally outlined in a logical framework and from the perspective of a program planner from a development agency. For additional indicators on SHN, program implementers can reference the Focusing Resources on Effective School Health (FRESH) Monitoring and Evaluation Thematic Indicators (28) and the WHO/UNESCO Global Standards for Health Promoting Schools and their Implementation Guidance (29) for examples of indicators across relevant intervention areas. In addition, the Global School-Based Student Health Survey (30) tracks health behaviors among students and the WHO Global Action for Measurement of Adolescent Health (GAMA) (31) consolidates and tracks health and policy indicators relevant for this cohort.

**Table 2:** Examples of school health and nutrition indicators by intervention.

**Intervention**	**Input indicators**	**Process and output indicators**	**Outcome indicators**
Vision screening	National poverty reduction strategy, human capital strategy, or other relevant strategy includes disability prevention	Number of primary and secondary schools that screen students for vision problems	% of students identified to have correctable vision loss that received readymade or low-cost spectacles
HPV vaccination	National vaccination program includes HPV vaccination for girls age 9–14 years, with a recommendation for school-based delivery	Number of participating schools that deliver two doses of the HPV vaccine to 80% of targeted female students	% increase of adolescent girls vaccinated with 2 doses of HPV vaccine by age 15 years
Intermittent iron and folic acid supplementation	National nutrition guidelines include a specific recommendation for school-based delivery of weekly IFA supplementation	Proportion of schools delivering weekly IFA supplementation to adolescent girls	% of adolescent girls aged 11–19 who received IFA supplements in the project area
School feeding	School feeding is included within the national poverty reduction strategy, human capital strategy, or equivalent strategy	Number of students attending schools which implement the health and nutrition program are fed one hot meal and one snack daily 190 days per year	% increase in number of school feeding days as percentage of actual school days in prior term
Deworming	Inclusion of helminth and schistosome control commodities in the basic package of school health interventions	Number of school-age children receiving anti-helminth treatment	% reduction in anemia and severe anemia among adolescents aged 10–19 years
Nutrition education and health promotion	National policies on the nutritional standards of food and beverages sold in school canteens are published	Number of schools with a safe and clean space that can be used for recess, sports, physical education, or other physical activity	% increase in number of adolescents participating in at least 60 min of physical activity per day during the past 7 days compared to baseline
Comprehensive sexuality education	Ministerial Order allowing adolescent girls to remain enrolled in school in the event of pregnancy or marriage	Percentage of secondary schools offering sexual and reproductive health services (information and/or contraceptive methods)	Number of additional institutions that have teachers trained to teach comprehensive sexuality education 2 years after baseline
Menstrual health and hygiene	National policies guarantee the provision of facilities and materials for adolescent girls and female teachers to manage menstrual health and hygiene safely and with dignity at school	Number of primary and secondary schools with separate latrines for girls to use	Number of districts (or other appropriate administrative unit) where 90 percent of public schools have access to safe water and sanitary facilities

*Most of the indicators included within this table are drawn from current and past World Bank projects as well as from the FRESH Monitoring and Evaluation Thematic Indicators (28). Note that impact indicators are not captured within the table, as the impact is often anticipated to occur beyond the life of a project. Examples of impact indicators include, reduced preventable deaths from cervical cancer and greater employment and earning opportunities. In the case where projects do not have indicators specific to school health and nutrition, the selection of indicators should best correspond to the results outcome desired*.

Program implementers must weigh several considerations when defining which indicators to measure and which data sources to utilize, frequency of data collection, roles for collecting and submitting data, and how to ensure the findings enable program adaptation. These include:

### Considerations Related to the Selection of Indicators

*1. Determine indicators along the results chain based on the intended final impact*

Program planners are encouraged to develop a theory of change to define the expected results among key target groups, and intervention components that will enable their achievement. This mapping will also be critical to identifying the relevant indicators that need to be measured (32), which will be heavily dependent on the intended final impact of the program. There is much debate around whether certain indicators are output, outcome, or impact indicators, but this will ultimately depend on the final outcomes expected in each program. For example, if the desired impact of an investment is to reduce adolescent pregnancy, then the delivery of comprehensive sexuality education to adolescents in secondary schools would be an outcome indicator on the pathway to get you to that impact. However, if increasing the access to and use of modern contraceptives among adolescent populations is the desired impact, then the outcome indicator could be related to the coverage of a family planning program that targets adolescents. In the context of time-bound investments in which school health and nutrition may be one activity within a larger investment, indicators should be selected carefully and represent not only inputs and outcomes but also program processes to track implementation, enable program adaptation, and subsequently increase the likelihood of achieving the intended objectives within the project period. Again, this is where the critical role of alignment with the program's logical framework comes to light.

*2. Clarify the details of how indicators will be measured and assessed, with special considerations for defining your target population*.

Equally important to selecting the indicators of interest is ensuring the ability to analyze data along key equity dimensions (such as gender and age groups), as success is dependent on the quality, adequacy, and the reach of the interventions. As with any SHN program that includes delivery of commodities to specific population groups, like school-based HPV vaccination programs for girls ages 9–14 years, determining the total number of targeted beneficiaries is critical. When targeting is meant to be age-based, program planners may consider calculating their targets using grade-based eligibility instead, as determining age is difficult in contexts where birth records are not routinely available, school registers are inaccurate, and/or census data is outdated (33).

*3. Reflect on and clarify who is implementing and monitoring the program*

The actors initiating and implementing SHN programs also influences the definition of indicators within each category of the logical framework. For example, for a government, an input-level indicator could be the number of supplies or commodities procured for program functioning, while for a donor agency, it may be the development of a SHN policy. National programs and monitoring generally operate under one ministry, while implementation may be led by technical bodies at the national or sub-national level, as is seen with school feeding (18, 19). It is important to note that SHN programming may be led by both state and non-state actors, thereby requiring considerable coordination and human resource costs to ensure consistency across operational frameworks as well as data collection and reporting throughout the period of program development and implementation.

Beyond the considerations about the program implementer, it is equally important to specify the actors who will support programmatic monitoring and reach agreement on the process for collecting data and reporting against each indicator. Data collection should be standardized across the program regardless of service delivery point to facilitate data aggregation and analysis (34). This is particularly important as multisector investments like school health and nutrition tend to engage representatives across ministries and organizations. Lack of clarity on these roles can create duplications and inconsistencies in reporting of key program monitoring indicators. In addition, capacity gaps related to programmatic monitoring and reporting similarly presents a common challenge.

### Considerations Related to Information Systems for Program Monitoring, and Frequency of Data Collection

*1. Identify available data sources and whether these require further strengthening*

Recognizing that national health management information systems may be too weak to be the sole source of data for programmatic monitoring, implementors can also consider complementary forms of data collection, such as through SMS or phone-based surveys and through innovative geo-enabled data collection mechanisms. Where needed, project funding can also include resources for support with data analysis, visualization, and use, or to strengthen the information system. To facilitate strengthened cross-sector collaboration and evidence-based decision making, data collected should routinely be shared, reviewed, and discussed between relevant sectors and institutions.

*2. Ensure an appropriate balance between minimum and ideal program monitoring and indicator selection standards to improve feasibility and minimize monitoring costs*.

Minimum standards are those which are necessary to achieve the desired outcome or to achieve country requirements, while ideal standards are more ambitious. In many cases, project teams might determine which indicators to prioritize based on what data collection systems are already in place or based on what can organically complement other efforts in the same space, rather than establishing siloed but more burdensome and costly approaches to collect an ideal indicator. As with any program, quality assurance mechanisms are essential for ensuring robust data collected against each indicator during collection, transfer, compilation, and analysis (35). This is especially true when routine program monitoring data is collected by different partners with different capacities and working at different levels.

*3. Explore opportunities for integrating monitoring into other school-based sources of information*.

Monitoring by development partners is often program specific and not necessarily aligned to government programs or data sources, which places a significant data reporting burden on implementing entities and misses an important opportunity to build monitoring capacity at the central and local levels. Therefore, where possible, program indicators should be aligned to those of national programs and/or programs implemented by other development partners, as they will already be collected through existing monitoring systems. Not only should indicators be aligned, but ideally so would data sources and frequency of data collection. For example, in countries with schools providing comprehensive sexuality education, integrating questions into annual, national, and standard assessments administered to test students' knowledge of key topics covered can be a useful and independent source of data to monitor the program's impact. This approach allows for standardized, routine and repeated learning assessments, which can generate independent and longer-term program feedback and can be used to assess intervention feasibility and effectiveness.

### Considerations Related to Feedback and Referral Mechanisms

*1. Ensure feedback mechanisms exist to enable program course correction*

Recognizing that SHN programs engage actors across sectors, it is important to build in a feedback loop that connects education and health information systems, especially in cases where the monitoring responsibility is with the education system (Figure 1, see Malawi example specifically). Data sources and reporting responsibility will thus vary based on the delivery model that is used, so clarity in these protocols and how the data will eventually reach the Ministry of Health, is essential. Protocols should clearly specify data sources, collection methods (including who collects and what monitoring tool is used), frequency, how routine monitoring data will be analyzed and used as it moves from one administrative level to another, and how it will be communicated, disseminated, and used for program- and implementation-related decision-making. This feedback loop should also flow back from the national to sub-national level for decisions around resource allocation and program course correction. Concerted efforts to build capacity in data management, analysis, and reporting will likely be needed at all levels of the system to ensure that this feedback loop functions properly.

*2. Identify how referrals from schools to facilities will be tracked and monitored*.

**Figure 3:**
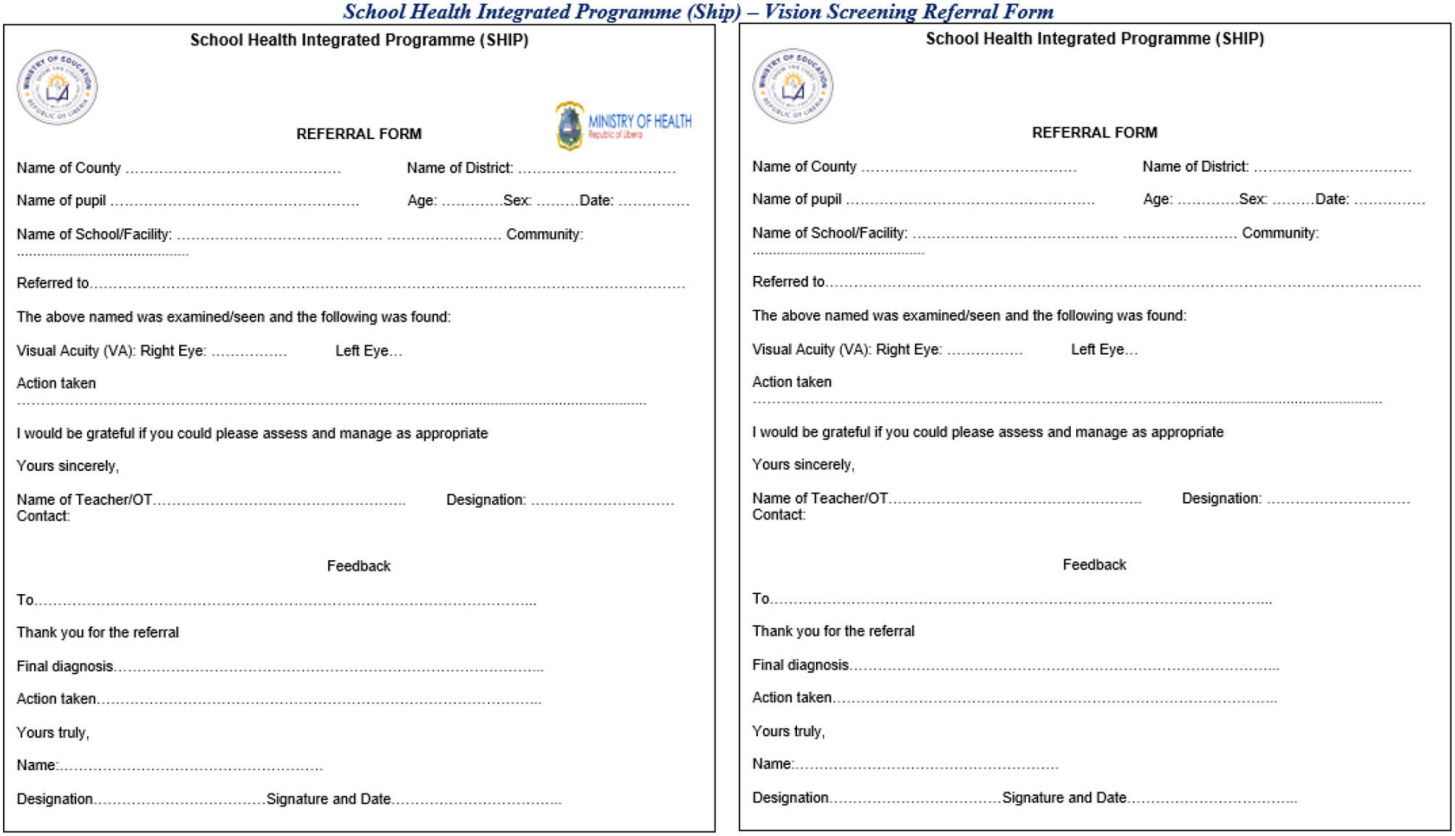
Example of referral forms used in Liberia for school-based deworming and vision screening.

Schools should have an established and well-disseminated referral mechanism between the school and health facilities and other appropriate institutions/agencies. Staff, be it health or education, who teach health and/or nutrition education should be equipped to refer students who present with symptoms and monitor whether the student received care, including establishing necessary linkages to support a student who faces a situation of abuse (ex. with local police, mental health counseling, etc.). It is important to ensure that there is a mechanism to track whether referred pupils were ultimately seen by a health or other relevant specialist (Figure 3). Linkages with the health system can also entail redistributing excess materials from SHN interventions to health facilities. School-based deworming campaigns, for example, often have agreements to redistribute unused tablets to health facilities and the number of redistributed tablets should be recorded as part of the deworming campaign.

## Discussion (INCL. Lessons Learned for Future Applications)

Program monitoring is both a management tool and a powerful feedback mechanism. It is best practice to integrate project monitoring from the earliest stages of project design, in alignment with the program's logical framework. The selection of indicators drives resource allocation toward the areas being assessed and incentivizes their implementation.

There are known gaps in the monitoring of SHN programs. At the global level, there is no repository of the health and nutrition status of children and adolescents nor of the breadth and coverage of SHN services provided in countries (36). Since coverage from school-based service delivery programs may not be captured within administrative surveys, SHN monitoring systems may need to rely on multiple systems to collate and monitor the relevant program indicators. While there is no internationally accepted standardized framework for the monitoring of SHN service delivery, there are a wide range of indicators that can be drawn upon and further contextualized to meet specific programmatic needs and activities.

There are key principles to develop a robust monitoring system that apply whether the service delivery is standalone or integrated with multiple services. In the context of school health and nutrition service delivery, multiple sectors will likely be engaged, and as such, each monitoring system will be unique to the programs, interventions, and setting. It is prudent that all engaged actors agree in advance on the process for collecting and transmitting data between levels and sectors, including, for example, interoperability between systems.

The COVID-19 pandemic, and the resulting prolonged school closures, has forced innovations in education, health, and nutrition service delivery mechanisms, and similarly, has necessitated varied approaches to monitoring the reach and quality of adapted interventions. There are emerging examples of how service providers have pivoted to reach these populations, with examples of innovative school meal distribution and tracking methodologies described above. The current pandemic shows the importance of developing and routinely updating emergency preparedness plans that outline contingency protocols for service delivery during school closures and localized disease outbreaks. These protocols should include guidance for which metrics to prioritize and which actors to task with collecting the information. In the case of prolonged school closures, community members could be called upon to prioritize indicators to monitor and to engage in data collection activities to inform adaptive measures (37).

Beyond the school closure period and as a country moves toward reopening, monitoring mechanisms may continue to need shifting and adaptation to respond to the new reality under which schools operate to comply with public health guidelines. For example, in the case of COVID-19, this includes coordination across sectors to implement mechanisms to monitor, report, and trace cases among students and teachers alike, monitoring and procurement of personal protective equipment and sanitation supplies to minimize spread within the school setting, and ability to track and respond to the broad spectrum of changing physical and mental health needs of adolescents as they return to schools after periods of extended closure. Many of the key principles outlined in this manuscript are increasingly relevant in such instances of sudden school closures, and again emphasize the need for rapid action, coordination, and innovation.

## Acknowledgment of Conceptual or Methodological Constraints

The authors acknowledge the relative paucity of country-specific information published on this topic. As such, the illustrative country examples and the key principles presented within this manuscript represent the best attempt and most complete information that the authors were able to collate through a desk review. The authors recognize that there is wide variation in approaches and compositions of SHN services within and across countries, and as such, it is impossible to be prescriptive around SHN monitoring methodology. Instead, the authors deemed it more efficient to guide program implementers with key principles for program monitoring.

## Data Availability Statement

The original contributions presented in the study are included in the article/supplementary material, further inquiries can be directed to the corresponding author.

## Author Contributions

LS and JR-B worked in tandem to equally contribute to the conceptual framing, research, writing, and editing of this manuscript. All authors contributed to the article and approved the submitted version.

## Conflict of Interest

The authors declare that the research was conducted in the absence of any commercial or financial relationships that could be construed as a potential conflict of interest.

## References

[1] Baltag V Pachyna A Hall J. Global overview of school health services: data from 102 countries. Heal Behav Policy Rev. (2015) 2:268–83. 10.14485/HBPR.2.4.4

[2] Bundy D Schultz L Sarr B Banham L Colenso P Drake L. The school as a platform for addressing health in middle childhood and adolescence. In: Bundy DAP, de Silva N, Horton S, Jamison DT, Patton G, editors. Disease Control Priorities, Third Edition (Volume 8): Child and Adolescent Health and Development. Washington, DC: IBRD/World Bank (2017). p. 269–85. 10.1596/978-1-4648-0423-6_ch20 30212136

[3] Baltag V Saewyc E. Pairing children with health services: the changing role of school health services in the twenty-first century. In: Cherry A, Baltag V, Dillon M, editors. International Handbook on Adolescent Health and Development: The Public Health Response. Cham: Springer (2017). p. 463–77. 10.1007/978-3-319-40743-2_24

[4] UNESCO. *Gross enrolment ratio by level of education* [Internet]. Available online at: http://data.uis.unesco.org/index.aspx?queryid=142 (accessed March 13, 2020).

[5] Reavley N Patton GC Sawyer SM Kennedy E Azzopardi P. Health and disease in adolescence. In: Bundy DAP, de Silva N, Horton S, Jamison DT, Patton GC, editors. Disease Control Priorities, Third Edition (Volume 8): Child and Adolescent Health and Development. 3rd ed. Washington, DC: World Bank Group (2017). p. 239–52. 10.1596/978-1-4648-0423-6_ch18 30212139

[6] Burbano C Ryckembusch D Fernandes M Mitchell A Drake L. Re-imagining school feeding: a high-return investment in human capital and local economies. In: Bundy DAP, de Silva N, Horton S, Jamison DT, Patton G, editors. Child and Adolescent Health and Development: Disease Control Priorities [Internet]. 8th ed. Washington, DC: World Bank Group (2018). p. Xiii–xxiii. Available online at: http://dcp-3.org/sites/default/files/resources/CAHD_eBook.pdf (accessed June 20, 2020).

[7] State of School Feeding Worldwide 2020. SAVING LIVES CHANGING LIVES. State of School Feeding Worldwide (2020).

[8] Patton G Temmerman M. Evidence and evidence gaps in adolescent health. J Adolesc Heal. (2016) 59:S1–3. 10.1016/j.jadohealth.2016.08.001 27664591 PMC5026676

[9] Maryland State Department of Education Maryland Department of Health. Maryland State School Health Services Guidelines: Management of Diabetes in Schools. Baltimore, MD (2017).

[10] Baltag V Levi M. Organizational models of school health services in the WHO European Region. J Heal Organ Manag. (2013) 27:733–46. 10.1108/JHOM-08-2011-0084 24422256

[11] Kroneman M Boerma W van de Berg M Groenewegen P de Jong J van Ginneken E. Netherlands: health system review 2016. Health Syst Transit. (2016) 18:1–239. 27467715

[12] Barnekow V Buijs G Clift S Jensen BB Paulus P Rivett D. Health-Promoting Schools: A Resource for Developing Indicators. International Planning Committee (2006).

[13] WHO Regional Office for Europe. Pairing children with health services: the results of a survey on school health services in the WHO European Region. (2010). p. 1–30. Available online at: http://www.euro.who.int/document/e93576.pdf (accessed September 8, 2020).

[14] Baltag V Stronski S Pattison D. School health services in Former Socialist Countries: case studies from Albania, Republic of Moldova, Tajikistan, and Ukraine. In: Dherry AL. Baltag V, Dillion ME, editors. International Handbook on Adolescent Health and Development: The Public Health Response. Geneva: World Health Organization (2016). p. 479–88. 10.1007/978-3-319-40743-2_25

[15] Khodjamurodov G Rechel B. Tajikistan: health system review. Health Syst Transit. (2010) 12:157. 21132994

[16] Ministry of Health Ministry of Education Science and Technology. Kenya National School-Based Deworming Programme Year 2 Report (2014).

[17] Ministry of Health UNICEF. Evaluation of the Weekly Iron and Folic Acid Supplementation. (2014) p. 1–83. Available online at: https://www.unicef.org/evaldatabase/files/Bhutan_Evaluation_of_the_Weekly_Iron_and_Folic_Acid_Supplementation_(WIFS)_Program-2004-2014.pdf (accessed May 7, 2020).

[18] Drake L Woolnough A Burbano C Bundy DAP. Partnership for Child Development. Global School Feeding Sourcebook: Lessons from 14 Countries. London: Imperial College Press (2016).

[19] Gelli A Espejo F. School feeding, moving from practice to policy: reflections on building sustainable monitoring and evaluation systems. Public Health Nutr. (2013) 16:995–9. 10.1017/S1368980012003989 22995677 PMC10271286

[20] Saito J Keosada N Tomokawa S Akiyama T Kaewviset S Nonaka D. Factors influencing the National School Health Policy implementation in Lao PDR: a multi-level case study. Health Promot Int. (2015) 30:843–54. 10.1093/heapro/dau016 24694681

[21] Ruel-Bergeron JC Hurley KM Kang Y Aburto N Farhikhtah A Dinucci A. Monitoring and evaluation design of Malawi's Right Foods at the Right Time nutrition program. Eval Prog Plann. (2019) 73:1–9. 10.1016/j.evalprogplan.2018.11.001 30453182

[22] UNESCO. Teacher Task Force calls to support 63 million teachers touched by the COVID-19 crisis [Internet] (2020). Available online at: https://en.unesco.org/news/teacher-task-force-calls-support-63-million-teachers-touched-covid-19-crisis (accessed May 18, 2020).

[23] The Global Women's Institute GAGE. Violence Against Adolescent Girls: Trends and Lessons for East Africa [Internet]. (2014). Available online at: https://www.unicef.org/publications/files/A_Statistical_Snapshot_of_Violence_Against_Adolescent_Girls.pdf (accessed May 7, 2020).

[24] Mayurasakorn K Pinsawas B Mongkolsucharitkul P Sranacharoenpong K Damapong S nga. School closure, COVID-19 and lunch programme: unprecedented undernutrition crisis in low-middle income countries. J Paediatr Child Health. (2020) 56:1013–7. 10.1111/jpc.15018 32619327 PMC7361388

[25] Save the Children. Save Our Education: Protect Every Child's Right to Learn in the COVID-19 Response and Recovery. London, UK (2020).

[26] WFP repackages efforts to reach hungry children as COVID-19 closes schools [Internet]. Devex (2020). Available online at: https://www.devex.com/news/wfp-repackages-efforts-to-reach-hungry-children-as-covid-19-closes-schools-96878 (accessed July 21, 2020).

[27] GCNF. Webinar: School Meals in the Time of COVID-19: Impact and Responses in India [Internet] (2020). Available online at: https://gcnf.org/covid/webinars/covid19-india-part1/ (accessed September 22, 2020).

[28] UNESCO. Monitoring and Evaluation Guidance for School Health Programs Thematic Indicators Supporting FRESH (Focusing Resources on Effective School Health) [Internet] (2014). Available online at: http://creativecommons.org/licenses/by-nc-sa/4.0/deed.en_US (accessed December 10, 2020).

[29] WHO UNESCO. Global Standards for Health Promoting Schools and their Implementation Guidance [Internet]. Geneva, Switzerland (2021). Available online at: https://www.who.int/maternal_child_adolescent/adolescence/global-standards-for-health-promoting-schools-who-unesco.pdf (accessed April 23, 2020).

[30] WHO. Global school-based student health survey (GSHS) [Internet]. Available online at: https://www.who.int/ncds/surveillance/gshs/en/ (accessed November 10, 2020).

[31] Guthold R Moller AB Azzopardi P Ba MG Fagan L Baltag V. The Global Action for Measurement of Adolescent Health (GAMA) initiative—rethinking adolescent metrics. J Adolesc Heal. (2019) 64:697–9. 10.1016/j.jadohealth.2019.03.008 31122505 PMC6531407

[32] Rossi PH Lipsey MW Henry GT. Evaluation: A Systematic Approach [Internet]. 8th ed. Thousand Oaks, CA: Sage Publications Inc (2019). p. 1–360. Available online at: https://us.sagepub.com/en-us/nam/evaluation/book243885 (accessed January 29, 2020).

[33] PATH LSHTM. HPV vaccine lessons learned and recommendations [Internet] (2016). Available online at: www.lshtm.ac.uk (accessed June, 2020).

[34] Partnership for Child Development. School Feeding Monitoring and Evaluation Toolkit [Internet] (2011). Available online at: http://hgsf-global.org/en/bank/downloads/doc_details/261-school-feeding-monitoring-and-evaluation-toolkit (accessed July 30, 2020).

[35] Görgens M Kusek JZ. Making Monitoring and Evaluations Systems Work: A Capacity Development Toolkit. Washington, DC: World Bank Group (2009). p. 1–530. 10.1596/978-0-8213-8186-1

[36] UNESCO. Stepping up effective school health and nutrition: A partnership for healthy learners and brighter futures [Internet]. Paris, France (2020). Available online at: www.hgsf-global.org/en/themes/shn (accessed November 11, 2020).

[37] Carvalho S Rossiter J Angrist N Hares S Silverman R. An Evidence Kit for Policymakers Planning for School Reopening and Recovery After COVID-19 [Internet]. Washington, DC (2020). Available online at: www.cgdev.org (accessed December 3, 2020).

